# Couples’ experiences with expanded carrier screening: evaluation of a university hospital screening offer

**DOI:** 10.1038/s41431-021-00923-9

**Published:** 2021-06-21

**Authors:** Ivy van Dijke, Phillis Lakeman, Naoual Sabiri, Hanna Rusticus, Cecile P. E. Ottenheim, Inge B. Mathijssen, Martina C. Cornel, Lidewij Henneman

**Affiliations:** 1 Center for Reproductive Medicine, Amsterdam Reproduction and Development Research Institute, Amsterdam UMC, University of Amsterdam, Amsterdam, the Netherlands; 2 Department of Clinical Genetics, Amsterdam Reproduction and Development Research Institute, Amsterdam UMC, Vrije Universiteit Amsterdam, Amsterdam, the Netherlands; 3 Department of Clinical Genetics, Amsterdam Reproduction and Development Research Institute, Amsterdam UMC, University of Amsterdam, Amsterdam, the Netherlands

**Keywords:** Genetic testing, Human behaviour

## Abstract

Preconception carrier screening offers couples the possibility to receive information about the risk of having a child with a recessive disorder. Since 2016, an expanded carrier screening (ECS) test for 50 severe autosomal recessive disorders has been available at Amsterdam Medical Center, a Dutch university hospital. This mixed-methods study evaluated the experiences of couples that participated in the carrier screening offer, including high-risk participants, as well as participants with a general population risk. All participants received genetic counselling, and pre- (*n* = 132) and post-test (*n* = 86) questionnaires and semi-structured interviews (*n* = 16) were administered. The most important reason to have ECS was to spare a future child a life with a severe disorder (47%). The majority of survey respondents made an informed decision (86%), as assessed by the Multidimensional Measure of Informed Choice. Among the 86 respondents, 27 individual carriers and no new carrier couples were identified. Turn-around time of the test results was considered too long and costs were perceived as too high. Overall, mean levels of anxiety were not clinically elevated. High-risk respondents (*n* = 89) and pregnant respondents (*n* = 13) experienced higher levels of anxiety before testing, which decreased after receiving the test result. Although not clinically significant, distress was on average higher for carriers compared to non-carriers (*p* < 0.0001). All respondents would opt for the test again, and 80.2% would recommend it to others. The results suggest that ECS should ideally be offered before pregnancy, to minimise anxiety. This study could inform current and future implementation initiatives of preconception ECS.

## Introduction

Carrier screening is used to investigate whether a couple has an increased risk of having children with a severe autosomal and X-linked recessive genetic disorder in order to facilitate reproductive decision making. Ideally, carrier screening is offered before pregnancy to allow for a maximum number of reproductive options, including preimplantation genetic testing (PGT) and prenatal diagnosis (PND).

Historically, screening initiatives were mainly ancestry based, addressing individuals from specific ethnic groups with a known increased risk for particular recessive inherited conditions. Due to the development of next-generation sequencing, screening for large panels of recessive conditions became possible at lower costs, resulting in carrier screening being increasingly offered to the general population, i.e., universal expanded carrier screening (ECS) [1, 2]. Several benefits of universal ECS compared to ancestry-based screening for high-risk populations have been proposed, such as equity of access to screening and the potential reduction of stigmatisation of ethnic groups [3]. However, disadvantages of ECS are the potential higher costs due to increased testing of broader panels, workload, and the possible emotional impact of test results [2]. Moreover, disability rights groups have expressed criticism towards the availability of ECS because of its tendency to negatively shape public opinion about disabilities [4, 5].

Worldwide, ECS panels are mainly offered by commercial companies, which have been criticised for using persuasive language, not providing complete information and offering only optional genetic counselling [6]. Increasingly, non-profit healthcare initiatives are emerging, yet large differences between initiatives and countries exist [7, 8] leading to questions about how ECS should be offered and by whom. Several expert bodies have been drafting recommendations on how ECS can be responsibly implemented, emphasising the importance of accurate and complete information provision, appropriate education of health professionals and the need for research into public perceptions on ECS and its psychological impact [1, 2, 9].

To our knowledge, the majority of literature assesses the hypothetical interest in ECS [10] and few studies have been conducted among users of ECS [11–14]. Research on the impact of carrier screening for a single autosomal recessive disease, cystic fibrosis (CF), has shown that identified carriers generally have no adverse long-term psychological consequences [15]. However, some studies showed that health perception was negatively influenced after screening, along with increased anxiety and inability to recall test results accurately [13, 16]. Moreover, it has been questioned whether people can make a sufficiently informed decision if there are multiple conditions in one panel, which can cause an information overload [2]. One study that investigated the impact of ECS showed that respondents with negative test results generally did not experience long-term negative emotional impact, and only reported heightened anxiety while waiting for the test results [13]. Additional studies have shown that the information from ECS could relieve uncertainty and anxiety [17], was of value to participants [11, 18] and led to informed reproductive decision making [19]. Reasons to have ECS among both high-risk groups and the general population need further investigation [10].

In the Netherlands, two university hospitals have developed preconception ECS tests for the general population [20, 21]. The Amsterdam University Medical Centers, location Academic Medical Center (AMC) Amsterdam has offered a test for 50 severe autosomal recessive disorders since 2016, and the University Medical Center Groningen offers a test for 70 autosomal recessive disorders.

In this study, we investigate experiences of ECS test participants at the AMC hospital Amsterdam, who agreed to participate in a survey or interview study, from both high-risk groups and the general population in terms of reasons to opt for the test, whether choices were informed, psychological well-being before and after the test, changes in reproductive intentions and satisfaction with the test.

## Materials and methods

### Study design

A mixed-methods parallel design was used to evaluate the Amsterdam ECS offer including a survey study using pre-test (Q1) and post-test questionnaires (Q2), and semi-structured interviews to gain an in-depth understanding of participants’ experiences [22]. Approval for this study was obtained from the AMC Medical Ethics Review Committee (W16_131#16.152).

### Sample population and setting

Couples or individuals interested in the ECS test applied for participation via online registration on the AMC website (https://www.dragerschapstesten.nl, accessed 13 March 2021) or were referred by a physician (high-risk couples only). The ECS participants, who also participated in the current study, all received pre-test genetic counselling at the AMC, supported by a leaflet and online information. Only one couple decided not to have the ECS test after counselling and did not participate in the survey study. The ECS test is available for all couples planning a pregnancy for a fee (€650 per test) and are reimbursed for high-risk couples by insurance, except for the ‘own risk’ excess (€385 per year). Participants were assigned as high-risk group if they had an increased risk of being a carrier (couple) for one or more of the 50 disorders based on a positive family history, consanguinity, ancestry and/or geographical background (i.e., people at risk for hemoglobinopathy, individuals living in a specific Dutch genetically isolated community (founder population), and those from Ashkenazi Jewish descent). Participants from the general population were assigned as general-risk group. Respondents could opt for parallel (both partners simultaneously) or sequential testing (one partner first, second partner only after the first partner tested positive).

### Survey and measurements

Q1 was administered after pre-test genetic counselling to 171 test participants (involving 69 couples and 33 individuals), between May 2016 and May 2018. Q2 was sent to participants’ home addresses after they received all the test results. The questionnaires were developed by a multidisciplinary research group consisting of health scientists and clinical geneticists and based on earlier studies [23]. Topics addressed were: (i) reasons to have the test, (ii) informed choice, (iii) recall and understanding of test results, (iv) psychological well-being (anxiety, worry, distress, health perception), (v) reproductive intentions and (vi) satisfaction (see Supplementary S1 for questionnaire items). Reasons for having the test were assessed by the question ‘What was the main reason to opt for the preconception carrier screening test for 50 hereditary diseases?’. Respondents were asked to select one answer from a list of nine reasons. Informed choice was measured based on the Multidimensional Measure of Informed Choice (MMIC) [24], which defines a choice as ‘informed’ when respondents had a positive attitude towards ECS, a good level of knowledge and took the test or had a negative attitude, good knowledge and declined the test. Attitude was measured using a 4-item semantic differential 5-point scale divided into three equal categories (positive, neutral and negative). Respondents with a neutral attitude were removed from the analysis based on literature [24, 25]. Knowledge was measured by eight questions. The cut-off for sufficient knowledge was set at 75% (6/8 questions), based on literature [25]. Recall of test results was assessed in Q2: ‘Do you remember the result of the carrier screening test for you and/or your partner?*’*. The answers were verified with the actual test result. Anxiety was measured with the Dutch 6-item Spielberger State-Trait Anxiety Inventory (STAI) scale [26] during Q1 and Q2. Scores can range from 20 to 80. A score >40 was considered clinically relevant [27]. Worry was assessed in Q2 ‘I felt worried while waiting for the test result’. The Impact of Event Scale (IES) subscale intrusion (the extent to which people relive feelings, dreams or experiences) was used to measure distress after screening test results in Q2 [28]. IES subscale scores range from 0 to 21. A score of >9 was considered a high level of intrusion [29]. Health perception was measured in Q2 by asking if respondents felt less healthy after receiving the test result. Reproductive intentions were assessed in Q1, asking whether respondents expected that the test result would help with making decisions about having children, and again in Q2, whether their decisions changed after the ECS results. Satisfaction was measured with regard to experiences with the ECS itself, whether people would opt for the test again and recommend it to others. Opinions on counselling and costs were assessed in Q1, and opinions on waiting time in Q2.

### Interviews

Participants were invited for an interview at random (*n* = 66). Semi-structured interviews were conducted over a period of 5 months in 2017 by one researcher [HR]. All interviewees signed informed consent forms. The interviews explored in-depth experiences with the ECS including reasons to have the test, psychological impact and satisfaction (Supplementary S2).

### Data analysis

For the questionnaire data, descriptive analysis was done to outline the respondents’ characteristics. Respondents were treated as individual subjects since each partner of a couple could have different perceptions [30]. *χ*^2^ tests and *t*-tests were done to investigate whether there were significant differences before (Q1) and after receiving ECS results (Q2) between different subgroups. Depending on the outcome variable or when the data were not normally distributed, Wilcoxon’s rank sum test was used. To assess differences between groups and associations of variables with higher STAI scores following Q2, a linear regression analysis using analysis of covariance with a correction for the pre-test STAI scores (Q1) was carried out. The beta coefficients and confidence intervals reflect to what extent STAI scores decreased or increased. *P* values <0.05 were considered to be significant. All analyses were performed using IBM SPSS version 24.0. Interview transcripts were processed for content analysis by two researchers independently [HR and IvD] using thematic analysis with the programme MAXQDA.

## Results

### Characteristics of respondents

Q1 was returned by 140/171 (82%) ECS test participants and Q2 by 94/138 (68%). Eight people were excluded from the analysis due to missing data, resulting in 132 respondents for Q1 (22 individuals and 55 couples), and 86 respondents for Q2 (16 individuals and 35 couples). The characteristics of 89 high-risk and 43 general-risk respondents who completed Q1 are presented in Table 1. A total of 16/66 (24%) invited individuals were interviewed, 11 females and 5 males; 4 interviewees had an a priori high risk of being a carrier.

**Table 1:** Survey respondents’ characteristics.

	High-risk group, *n* = 89	General-risk group, *n* = 43	Total, *n* = 132
Sex, n (%)			
Female	51 (57.3)	22 (51.2)	73 (55.3)
Male	38 (42.7)	21 (48.8)	59 (44.7)
Age in years, mean (SD)			
Female	30.1 (4.4)	33.3 (3.9)	31.0 (4.5)
Male	34.4 (7.8)	35.7 (5.1)	34.8 (6.7)
Ethnicity^a^, n (%) missing 2			
Dutch	58 (65.2)	36 (87.8)	94 (72.3)
Other western	6 (6.7)	2 (4.9)	8 (6.2)
Non-western	25 (28.1)	3 (7.3)	28 (21.5)
Education^b^, n (%), missing 2			
Low	2 (2.2)	1 (2.4)	3 (2.3)
Intermediate	20 (22.5)	3 (7.3)	23 (17.7)
High	67 (75.3)	37 (90.2)	104 (80.0)
Religiously active^c^, n (%), missing 3	41 (46.5)	5 (12.1)	46 (35.7)
Have child(ren), n (%), missing 2	26 (29.9)	10 (23.3)	36 (27.7)
Relationship status, n (%), missing 1			
Married or cohabiting	78 (87.7)	41 (97.6)	119 (90.8)
Single	8 (9.0)	1 (2.4)	9 (6.9)
Other relationship^d^	3 (3.4)	–	3 (2.3)
Pregnant (partner or self) at time of testing^e^, missing 2	8 (9.2)	5 (11.6)	13 (10.0)
A priori high-risk^f^	89 (67.4)	**–**	89 (67.4)
Positive family history^g^	30 (33.7)	–	30 (33.7)
Consanguinity	26 (29.2)	**–**	26 (29.2)
Ancestry			
Genetically isolated community	8 (9.0)	–	8 (9.0)
Ashkenazi Jewish	13 (14.6)	**–**	13 (14.6)
Hemoglobinopathy	13 (14.6)	–	13 (14.6)
Applied for ECS consultation, missing 5			
Actively signed up through website	25 (29.8)	40 (93.0)	65 (51.2)
Referred by a doctor	59 (70.2)	3 (7.0)	62 (48.8)

*ECS* expanded carrier screening, *SD* standard deviation.

^a^Based on Central Bureau of Statistics Netherlands definition.

^b^Low: elementary school, lower level of secondary school, lower vocational training; Medium: higher level of secondary school, intermediate vocational training, High: high vocational training, university.

^c^Religions included: Islam (*n =*16), Roman Catholic (*n =*16), Judaism (*n =*8), Protestant (*n =*4), Buddhist (*n=*1) and other religion (*n =*1).

^d^Engaged (*n =*1), in a relationship not living together (*n =*2).

^e^In the high-risk group 2 couples and 4 individual respondents indicated to be pregnant. In the general-risk group 2 couples and 1 individual respondent indicated to be pregnant.

^f^A priori high risk: of being a carrier or carrier couple. Respondents could have multiple medical indications.

^g^The familial disorders were: Alpers disease (*n =*2), Batten’s disease (*n =*2), Cystic fibrosis (*n =*8), Krabbe’s disease (*n =*6), Pompe’s disease (*n =*4), Spinal muscular atrophy (*n =*8).

### Reasons to have the test

The most important reason to have the test for both the high-risk and the general-risk group was to spare the future child a life with a severe disorder, 50.6% and 43.9% respectively (Table 2). Overall, 12.5% reported they were afraid they would regret it if they chose not to have the test. The least important reason to have the test for the high-risk group was to prepare for a child with one of the 50 disorders (2.3%). In the interviews, some participants mentioned that they chose to have testing because they wanted to avoid a difficult life for their child or did not want a (or another) child with a severe disorder: *“I have a child with some issues […]. We thought let’s do it [ECS]. So, I was one of those people who did not do the test out of curiosity, we did the test to exclude that it [having an affected child] would happen again.”* (Man, high-risk group, #10). One woman mentioned that being aware of ECS and not opting for it would result in guilty feelings if a child with a disorder was born: “*It may be a bit neurotic, but if I know that such a test exists, and I can do it, and I’ve got the money for it, then… If the baby would have a disorder and I would not have done the test, then I would feel guilty. That’s maybe a bit strange, but it played a role.”* (Woman, general-risk group, #5).

**Table 2:** Main reasons for respondents to have the preconception expanded carrier screening test.

Reasons	High-risk group, *n* = 89, *n* (%)	General-risk group, *n* = 43, *n* (%)	Total, *n* = 128, *n* (%)
I want to spare my child a life with a severe disorder	44 (50.6)	18 (43.9)	62 (53.1)
To avoid having a child with one of the disorders	19 (21.8)	17 (41.5)	36 (28.1)
Fear to regret afterwards when I do not have a test	10 (11.5)	6 (14.6)	16 (12.5)
Perceiving a high risk of being a carrier	15 (17.2)	–	15 (11.7)
Perceiving a high risk of having a child with one of the disorders	12 (13.8)	1 (2.4)	13 (10.2)
On the advice of others, namely…^a^	7 (8.0)	–	7 (5.5)
My partner wants it	4 (4.6)	1 (2.4)	5 (3.9)
For my own children (if they want children)	5 (5.7)	–	5 (3.9)
To prepare for a child with one of the disorders	2 (2.3)	–	2 (1.6)
Other reasons^b^	5 (5.7)	4 (9.8)	9 (7.0)

Percentages do not add up to 100% because respondents could fill in more than one reason. In each group there were *n* = 2 missing.

^a^General practitioner (*n =*3), parents (*n =*2), medical specialist at fertility clinic (*n =*1), clinical geneticist (*n =*1).

^b^Consanguinity (*n =*3), interested in knowing risk (*n =*3), (deceased) child with one of the 50 diseases (*n =*2), test is obligatory in other countries (*n =*1).

### Informed choice

Knowledge levels were significantly higher within the general-risk group: 97.7% had sufficient knowledge, compared to 83.1% of the respondents in the high-risk group (*p* = 0.017). Overall, 98% had a positive attitude towards having the ECS. Informed choice analysis showed that 86% of the respondents made an informed decision, which was not significantly different between the high-risk (81.5%) and general-risk group (94.3%) (*p* = 0.81). Uninformed choice was mostly explained by having poor knowledge (Table 3).

**Table 3:** Informed and uninformed choice for high-risk and general-risk respondents.

	Knowledge	Attitude	Uptake^b^	High-risk group (*n* = 65) (%)	General-risk group (*n* = 35) (%)	Total (%)
Informed^a^ choice	Good	Positive	Yes	81.5^c^	94.3^c^	86
Uninformed choice	Good	Negative	Yes	1	–	1
	Poor	Positive	Yes	17.5	5.7	13
	Poor	Negative	Yes	–	–	–

^a^An informed choice was made when respondents had a positive attitude towards expanded carrier screening, a good level of knowledge (75% correct answers) and took the test.

^b^All respondents agreed to have the test.

^c^Respondents with ‘neutral attitudes’ (*n* = 25) and missing on this variable (*n* = 6) were excluded from the analysis, based on van den Berg et al. [24].

### Recall and understanding of test results

Among the high-risk and general-risk respondents, 61.2% (30/49) and 75.6% (28/37) opted for sequential testing, respectively. The other respondents opted for parallel testing. All participants received full disclosure of their individual ECS test results. No new carrier couples were identified among the survey respondents, 7 couples already knew they were a carrier couple because they already had an affected child, and 27 carriers were identified (Supplementary Tables S4 and S5). Of the carriers, 92.6% (25/27) correctly recalled their own test result and that of their partner. One couple falsely indicated that they were a carrier couple of one of the 50 conditions included in the ECS, while they were carriers of another disorder not included in the test. Of the carriers, 37% (10/27) falsely reported that they had no chance of having a child with one of the 50 disorders from the test while there is always a residual risk. For those not identified as carriers (‘non-carriers’) and not tested respondents, this was 27.1% (16/59). After testing, overall knowledge slightly increased with a mean sum score of 6.97 [SD 1.26] at Q1 to 7.37 [SD 1.14] at Q2, although this difference was not significant (*p* = 0.233).

### Impact on psychological well-being

**Fig. 1:**
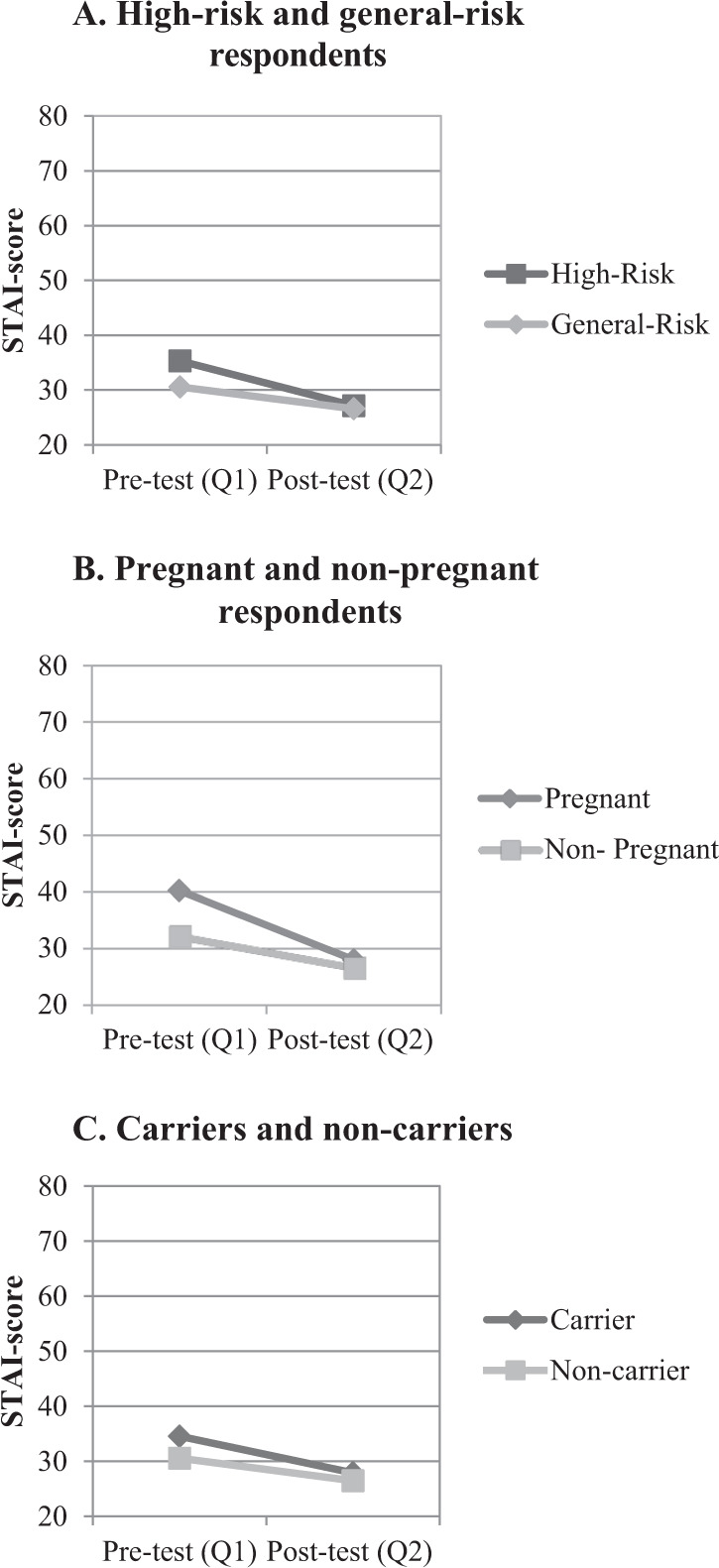
Mean Spielberger State-Trait Anxiety Inventory (STAI) scores for different groups over time. Scores (range 20–80) before the test (Q1) and post-test results (Q2) for high-risk and general-risk respondents (**A**), pregnant and non-pregnant respondents (**B**) and for carriers and non-carriers/not tested respondents (**C**). A score >40 is considered as clinically significant.

Figure 1 shows mean STAI scores for the different groups. Overall, mean anxiety scores were not clinically elevated. The high-risk group had higher anxiety levels 35.3 [SD 10.8] before receiving the test results compared to the general-risk group 30.5 [SD 10.1] (*p* = 0.03). Pregnant respondents had significantly higher anxiety 40.3 [SD 12.7] before the test result compared to non-pregnant respondents 32.3 [SD 10.4] (*p* = 0.01). Before testing, clinically significant STAI scores (>40) were found for 28/129 (21.7%) respondents [range 43.3–66.5], of which 23 were high-risk couples and 4 were pregnant. Overall mean STAI scores of respondents significantly decreased after receiving test results from 33.2 [SD 10.9] at Q1, to 26.9 [SD 8.9] at Q2 (*p* < 0.0001). There was no significant difference in anxiety between carriers 27.9 [SD 9.0] and non-carriers 26.49 [SD 8.9] after receiving the test result (*p* = 0.214). At Q2, 6/86 respondents (6.9%) showed clinically elevated STAI scores [range 43.3–66.5], of which one respondent was pregnant and three were carriers. Multiple linear regression analysis shows that respondents who made an uninformed choice concerning the test had a significantly higher mean STAI score at Q2 compared to those who did not, corrected for other variables (Table 4). Of the respondents, 35 (26.5%) indicated they were worried while waiting for the test results. Two respondents indicated that they sought additional information about the relevant conditions while waiting for their partners test results, which increased their stress levels. Some interviewees reported that they felt relieved when receiving their, or their partner’s, test results: *“When she said it was negative, I really felt such a big relief. I didn’t even know I was so stressed about it. But it turned out that I was thinking about it a lot, unconsciously. Because I was so relieved when hearing my husband was not a carrier of CF.”* (Woman, general-risk group, #2).

**Table 4:** Variables that correlate with higher STAI scores after the test result.

Variables	*β* (95% CI)	*p* value
A priori high risk^a^	1.857 (5.41 to –1.69)	0.914
Pregnant^b^	1.93 (–3.76 to 7.03)	0.368
Having children^c^	–1.47 (–5.47 to 2.53)	0.466
Sex^d^	–0.269 (–3.83 to 3.29)	0.881
Uninformed choice^e^	8.606 (1.12 to 16.08)	**0.025**
Carrier^f^	0.491 (–3.17 to 4.15)	0.791

Bold values indicate statistical significance *p* < 0.05.

^a^Adjusted for baseline score STAI (Q1).

^b^Adjusted for baseline score STAI (Q1) and being a priori high risk.

^c^Adjusted for baseline score STAI (Q1), being a priori high risk and being pregnant.

^d^Adjusted for baseline score STAI (Q1), being a priori high risk, being pregnant and having children.

^e^Adjusted for baseline score STAI (Q1), being a priori high risk, being pregnant, having children and being male.

^f^Adjusted for baseline score STAI (Q1), being a priori high risk, being pregnant, having children, being male and making an uninformed choice.

Distress at Q2 (IES-intrusion) was significantly higher for carriers compared to non-carriers, with a mean score 4.1 [SD 4.4] and 1.3 [SD 2.3], respectively (p < 0.0001). Six of the 27 carriers and one non-carrier (who was pregnant) had clinically significant levels of distress [(>9) range 10–13]. Of these six carriers, three had affected children and one had a deceased child with a recessive disease. After receiving the test result (Q2) none of the respondents, including all carriers, reported that they felt less healthy.

### Impact on reproductive intentions

At Q1, 114/132 (86.4%) respondents indicated that they would opt for PND if they turned out to be a carrier couple, 100/132 (75.8%) would consider termination of pregnancy if the child would be affected and 105/132 (79.5%) would like to have more information concerning PGT. For 19/86 (22.1%) respondents, reproductive plans changed after receiving the test results (Q2): two respondents (one carrier and one non-carrier) had doubts about having another child, one respondent wanted more children, and 16 respondents said they were more determined to have children. In the interviews, it was mentioned that the test could offer reassurance to start planning the pregnancy in case of a negative (favourable) result. *“He said: well we are both carriers of 0 diseases, then I said: do you realize what you said? We can start! [to conceive].”* (Woman, general-risk group, #11).

### Satisfaction

All respondents indicated that they would have the test again, and 80.2% would recommend the test to others, 12.8% did not know if they would recommend it and 7% would not recommend it. Reasons not to recommend the test to others included ‘I believe everyone should decide this themselves’ and ‘It is quite expensive so it depends on the financial situation of the person’. Almost half (49.6%) considered the costs of the test too high. Moreover, interviewees mentioned that a reason in favour of sequential testing was to save costs (potentially only one partner needs a test). Some interviewees believed that the high costs of the test could create inequality in access: *“I think it is good that the test [ECS] is available, however, you can ask yourself: is it not only available for people with sufficient resources? So, what exactly is the target group? People who are often highly educated and know that the test exists.”* (Woman, high-risk group, #15). The reported waiting time for (combined) results was generally seven weeks; 43.0% (37/86) of the respondents considered the waiting time too long. This was similar for respondents who opted for sequential or parallel testing. The vast majority of respondents (*n* = 114, 86.4%) considered it essential that people receive face-to-face pre-test counselling. Others believed that information can also be provided online (*n* = 10, 7.6%) or with a leaflet (*n* = 6, 4.5%). Moreover, interviewees indicated that narrative stories and experiences provided on the website could be informative for couples when deciding to have the test or not.

## Discussion

This is one of the first studies to evaluate experiences with an ECS test in a non-commercial hospital setting from the perspective of test participants. Our results show that most participants made an informed choice, experienced no or limited negative impact on psychological well-being and were satisfied with the test despite considering the cost of the test too high.

The most important reason for participants to have the test was to spare a future child a life with a severe hereditary disease. This is in line with previous survey studies assessing the hypothetical interest among potential users of ECS in the Netherlands [20, 21]. However, in those studies, the second most important reason to opt for the test was to prepare yourself for having a child with a severe disease [20, 21], while in our study only 2.3% considered this an important reason. This difference could be explained by the relatively high number of high-risk couples in our study who might have already experienced the burden of having a child with one of the 50 disorders [31].

Previously, concerns were raised that the expansion of the number of disorders in the test-panels, in addition to the growing number of reproductive options, could undermine couples’ informed decision making [2, 32]. In our study, a high percentage (86%) of respondents made an informed choice, which could be the result of the extensive pre-test genetic counselling that was provided. In literature, informed choice for population reproductive genetic screening initiatives ranged from 27 to 51% [33]. This discrepancy could be due to the variety of contexts in which MMIC was measured, to differences in the definition ‘good knowledge’ or differences in educational level that may be an explanation for the high levels of informed choice in this study. Most respondents in our study correctly recalled their test results. However, in line with other studies on single disorders [15] and smaller gene panels [23] respondents tended to misunderstand the implications of the residual risk of a screen-negative test, although the actual residual risks in general are low for the tested couples. This stresses the importance of adequate pre-and post-test counselling, as was mentioned before in the European Society of Human Genetics recommendations [2]. One possible solution to avoid information overload during counselling could be to offer generic consent. With generic consent, conditions and implications are explained more generally, such as the possibility to be carrier of a condition that is accompanied with a severe intellectual deficit, instead of counselling about all the possible conditions individually [34]. Moreover, when, in the future, ECS is offered as part of a population screening programme, face-to-face pre-test counselling by clinical geneticists only, as was done in this study, is not likely to be feasible.

Overall, mean levels of anxiety were not clinically elevated. Anxiety levels were higher before than after ECS results, which is in line with previous studies for one disorder [15] or a smaller panel [23]. Pregnant participants had relatively higher anxiety levels, which was in accordance with a recent ECS study among pregnant and non-pregnant women in China, in which higher anxiety levels were reported for pregnant respondents (and their partners) [14]. This confirms that ECS should preferably be offered before instead of during pregnancy [2, 35]. Moreover, our study shows that respondents who have made an informed choice had significantly lower levels of anxiety after testing, which emphasises the importance of informed decision making. Overall distress levels were not clinically significant, although for six carriers distress levels were high. This supports the importance of post-test counselling, especially for people with positive family history, to provide guidance on future reproductive choice [11].

No new carrier couples were identified among the survey respondents. Some respondents expected that the ECS would help them decide about having children and indicated that their wish to have children became stronger after the test. A review on reproductive decision making of couples at risk showed that most couples would opt for PGT or PND with possible pregnancy termination following their ECS result [19], similar to our data. Generally, respondents were satisfied with the test; all respondents indicated that they would do the test again and 80.2% would recommend the test to others, which is in line with a previous Dutch study in which satisfaction with CF carrier screening was assessed [36]. Almost half of the participants considered the costs of the test too high, even though it was reimbursed for the high-risk group. Earlier, it was shown that most individuals from the general public were prepared to pay €75 [21], and only 3% were willing to pay €500–1000 [20]. Attention should be paid to equal access when people have to pay for the test out-of-pocket, and reimbursement for those who cannot afford it should be considered [4]. Moreover, it would be interesting to investigate whether responses are different when the test would be offered free of charge.

There has been discussion in literature on whether it is better to give individual test results to couples, or couple-based test results where only results are disclosed if both partners are carriers for the same disorder (carrier couple). Although this study did not investigate respondents’ preferences towards test disclosure, literature shows that users generally prefer full disclosure of individual test results over couple-based results [37, 38]. A hypothetical survey study however showed that a majority of respondents had no objection towards receiving couple-results only. The latter is considered a more sustainable scenario in a public healthcare setting due to lower costs and workload [39]. Moreover, in order to avoid undue psychological impact, a couple-based approach could be more suitable [40].

### Strengths and limitations

This study assessed the experiences of participants having an ECS test in a healthcare setting using a mixed-methods design. Moreover, this study assessed perspectives of both high-risk groups and the general population. The results should, however, be interpreted with caution in terms of generalisability because the number of returned questionnaires after the test result was limited (*n* = 86), and most of the participants were highly educated. Although ECS allows testing regardless of risk, the majority of the participants in our study had a high a priori risk. This finding is similar to a study by Holtkamp et al. where an online direct-to-consumer test for CF intended for the general population was evaluated and mainly used by people with a positive family history [41]. Another limitation is that there is no ‘gold standard’ to measure informed choice [33], and couples’ deliberation for testing was not assessed. Moreover, we do not know the reasons of test-decliners. No new carrier couples were identified among respondents; therefore, the extent to which a positive carrier couple result impacts couples’ psychological well-being and reproductive decision making warrants further research.

## Conclusion

This is one of the first studies to evaluate the experiences of participants with an ECS test in a non-commercial setting. This study showed that both high-risk and general-risk participants were satisfied with having an ECS test for 50 severe autosomal recessive disorders. Genetic counselling was regarded as valuable. However, waiting time for results was considered too long and costs of the test too high. To increase accessibility, out-of-pocket costs ideally should be reduced. The majority of respondents made an informed decision, suggesting that the counselling and information protocols at the AMC worked well for this highly educated group. Adverse impacts on psychological well-being were limited, although our findings support that offering ECS before—instead of during—pregnancy can avoid anxiety among pregnant couples. Moreover, some carriers showed distress that could possibly be minimised by only disclosing couple-based test results. The results of this study could be relevant for the implementation of initiatives on preconception ECS.

## Supplementary information

Supplemental Material
