# Supplementary material for: Couples’ experiences with expanded carrier screening: evaluation of a university hospital screening offer
Source: Eur J Hum Genet. 2021 Jun 21;29(8):1252–8. doi: 10.1038/s41431-021-00923-9 (PMC8384865; doi:10.1038/s41431-021-00923-9)
Supplement: Supplementary file 1 — Supplemental Material [file 41431_2021_923_MOESM1_ESM.pdf]

Supplemental Information

Supplement S1

Topics addressed in Questionnaire Q1 (pre-test) and Questionnaire Q2 (post-test)

**Topic**	**Questionnaire**	**Question**
Reasons to have the test (i)	Q1	What was the most important reason for having the carrier test? (tick one box from a list of 9 reasons with the option to add other reason)
Informed choice (ii):		
Uptake	Q1	Whether respondents agreed to have the test
Knowledge	Q1 and Q2	Eight knowledge questions, see Supplement S3
Attitude	Q1	Having the carrier test for 50 serious hereditary diseases for me is [Negative-Positive, Difficult- Easy, Frightening – Not- frightening, Reassuring – Not-reassuring (5-point semantic scale)
Recall and understanding of test results (iii)	Q2	Do you remember the results of the carrier test of you and your partner? (Each participant was asked to answer if they and/or their partner is a carrier and for which condition; not a carrier; had not been tested)
Psychological well-being (iv):		
Anxiety (STAI)[26]	Q1 and Q2	Six statements that evaluate how respondents feel "right now"/at this moment, such as *I feel at ease*, with four answer categories 1 = not at all, 2 = somewhat, 3 = moderately and 4 = very much so
Worry	Q2	-I was worried waiting for my test results.<br>-I am worried now about the result of the carrier status test. (answer options: disagree, partly disagree, neutral, partly agree, agree)
Distress (IES)[29]	Q2	The occurrence of an event is scored as 1=not at all, 2=rarely, 3=sometimes, 4=often. Total of seven items/statements; how often did 'statement such as *I thought about it without wanting to* apply to you in the past 7 days' (subscale intrusion)
Health perception	Q2	I feel less healthy after hearing the test results (disagree, partly disagree, neutral, partly agree, agree)
Reproductive intention (v)	Q1 and Q2	- The results of the carrier test could help me in the future in making decisions about having children

		- I would not have (anymore) children if my partner and I were both carriers of the same condition<br>- I would opt for prenatal diagnosis if my partner and I were both carriers of the same conditions<br>- I would consider termination of pregnancy if the unborn child was affected with one of the 50 disorders<br>- I would like more information about an IVF treatment with embryo selection (pre-implantation genetic diagnosis), if my partner and I were both carriers of the same condition<br>(All above questions had the following answer categories: disagree, partly disagree, neutral, partly agree, agree)<br>-Did the test results change your ideas about having children? (Yes/No/ If yes, why?)
Satisfaction (vi)	Q1 and Q2	-If I had to decide again, I would participate again (If no, why not?)<br>-Would you recommend the screening to other people? (Yes/No/I don't know)<br>-I consider the costs of the test as too high (disagree, partly disagree, neutral, partly agree, agree)<br>-I received sufficient answers to my questions during the information meeting. (disagree, partly disagree, neutral, partly agree, agree)<br>- Do you think it is necessary that couples who want to have a carrier status test always have an interview first? (Yes: to check whether people understand enough, or to get people to ask questions, or because […]. No: this is also only possible with a leaflet, or this is also possible with information online (via the internet), or because […].<br>-I felt worried while waiting for the test result (disagree, partly disagree, neutral, partly agree, agree)

Abbreviations: STAI=Spielberger State-Trait Anxiety Inventory, IES=Impact of Event Scale, IVF= In vitro fertilization

Supplement S2

Interview guide

Topic list

Pre-test result experiences:

- General attitude toward genetic testing
- Application and information beforehand
- Expectations concerning test procedure
- Reasons to have the test

Counselling experience

- General views towards counselling
- Views on information provision

Waiting period and post-test result experience

- Impact of waiting time
- Communication of test result
- Meaning of test result
- Overall satisfaction

Supplement S3

Table S3. Percentages of correct answers per knowledge question pre (Q1) and post (Q2) test result

Question	% Correct answers **Q1 pre-test result**	% Correct answers **Q2 post-test result**
1. Even if such a disease did not previously occur in the family, couples could still have a child with one (or more) of these 50 disorders (=correct)	93.2	94.2
2. The expanded carrier screening test is only intended for people with a family history of a hereditary disease (=incorrect)	91.7	94.2
3. A carrier of one of these 50 diseases can develop health problems at a later age due to this carrier status (=incorrect)	72.7	88.4
4. Partners who are both carriers of the same disease from the test have a 25% (1 in 4) chance of having a child with this disease in each pregnancy (=correct)	96.2	97.7
5. Even if one of the partners is a carrier of a disease from the test, couples have a greatly increased risk of having a child with that disease (=incorrect)	88.6	93.0
6. Suppose: A man is a carrier of one disease and his partner is a carrier of another disease. The chance of a child with one of these diseases is greatly increased (=incorrect)	87.9	94.2
7. If both partners are carriers of the same disease, they can have an examination during pregnancy whether the unborn child has the disease (=correct)	90.9	91.9
8. If nothing is found with this test, there is still a very small chance that a child will be born with a disease from the test (=correct)	75.8	83.7

Supplement S4

Table S4. Carrier test result of respondents with positive test-results (n=27/86)

Number of (likely) pathogenic variants	Number of respondents (%)	Disorder
1	22 (81.5)	Spinal Muscular Atrophy (n=5), Cystic Fibrosis (n=2), Metabolic disorder (n=2), Ataxia with vitamin E deficiency (n=1), Autosomal recessive spastic ataxia type Charlevoix-Saguenay (n=1), Batten disease (n=1), Citrullinemia (n=1), Gaucher type 1[a] (n=1), MLD (n=1), Neuronal ceroid lipofuscinosis, type 2 (n=1), Niemann-Pick disease (n=2), Fetal Akinesia Deformation Sequence Syndrome (n=1), POLG-related disorder (n=1), Ponto cerebellar hypoplasia type 2 (n=1), Sanfilippo (n=1)
2	3 (11.1)	Cystic Fibrosis/Krabbe disease (n=1), Krabbe disease/Pompe disease (n=1), Spinal Muscular Atrophy/Sanfilippo (n=1)
3	1 (3.7)	Krabbe disease/Zellweger syndrome/Niemann-Picks disease (n=1)
4	1 (3.7)	Cystic Fibrosis/Sanfilippo/Glycogen Storage Disease type 1/Batten disease (n=1)

[a] Respondents with Ashkenazi Jewish descent could optionally be tested for this disease (https://www.dragerschapstesten.nl , accessed 13 March 2021)

Supplement S5

Table S5. Distribution of carriers and non-carriers in the high-risk group and general-risk group, as reported by respondents who completed Q2

	High-risk group, n	General-risk group, n	Total, n
**Carrier**	19	8	27
**Non-carrier**[a]	22	21	43
**Not tested**[b]	8	8	16
**Total**	49	37	86

[a] Respondents who were not identified as a carrier.

[b] Partner not identified as a carrier in sequential testing, therefore not tested.
